# Cancer care in Singapore

**DOI:** 10.2349/biij.4.3.e38

**Published:** 2008-07-01

**Authors:** J Tey, S Baggarley, KM Lee

**Affiliations:** The Cancer Institute at National University Hospital, Singapore

**Keywords:** Cancer care, Singapore

## Abstract

Singapore is a small country, but it is ideally and centrally located to conveniently serve not only its population but also patients from the surrounding regions. It’s economy is sufficiently strong to maintain highly sophisticated and expensive equipment to manage a high level of healthcare, including oncology services.

Cancer incidences in Singapore are on an upward trend based on the report of the Singapore Cancer Registry for the period of 2001-2005. Cancer is the number one cause of death in Singapore. The three most common cancers for males in Singapore, in decreasing occurrences, are colorectal, lung, and prostate. For females, the three most common cancers are breast, colorectal, lung cancers. Technological advances and advances in anti-cancer drugs have transformed cancer management leading to improved outcomes worldwide and in Singapore as well. The epidemiology and management of these common cancers in Singapore are presented. While Singapore presently has five radiotherapy centres (3 public, 2 private) to service its population of 4.5 million and regional needs, the government has plans to expand its radiotherapy services to accommodate the aging population and the rising expectations of increasingly affluent cancer patients seeking advanced cancer care. The current and future initiatives spearheaded by Singapore to achieve excellence in this aspect are discussed.

## EPIDEMIOLOGY

Cancer, being the most common cause of death in Singapore, accounts for 30% of all deaths annually. Epidemiological data is continuously being collected at the Singapore Cancer Registry, and data is obtained via notifications by the physicians, pathology records, hospital records and death certificates [1]. Table 1 shows the number of incident cancers by year of diagnosis from 2001 to 2005. In general, there was an upward trend in the number of incident cancers by year of diagnosis. Table 2 shows the number of incident cancer by gender in the same time period. Crude incidence rates for total male and female cancer patients in the same time period were 235.7 and 248.5 per 100,000 Singapore resident population respectively.

**Table 1:** Number of incident cancers by year of diagnosis, 2001-2005.

**Year of diagnosis**	2001	2002	2003	2004	2005	2001-2005
**No. of notifications**	8048	8315	8151	8917	8915	42,346

**Table 2:** Incidence of cancer by gender, 2001-2005

**Gender**	**Number**	**%**	CR*	ASR**
Male	20,532	48.5	235.7	226.5
Female	21,814	51.5	248.5	201.9

* CR Crude rate per 100,000 per year

** ASR Age standardised rate per 100,000 per yea. ASR Derived by the direct method using the ‘World Population’.

Tables 3 and 4 show the top ten most frequent cancers in males and females for the period 2001-2005. Although colorectal and breast carcinoma were the most common carcinomas respectively in males and females, lung, colorectal and liver cancers were the top 3 causes of cancer mortality in males, while breast, lung and colorectal cancers were the top 3 causes of cancer mortality in females.

**Table 3:** Ten most frequent cancers in males, 2001-2005

**Rank**	**Site**	**No.**	**%**	CR*	ASR**
1	Colo-rectum	3665	17.9	42.1	40.4
2	Lung	3657	17.8	42.0	41.1
3	Prostate	1773	8.6	20.4	20.7
4	Liver	1660	8.1	19.1	18.4
5	Stomach	1366	6.7	15.7	15.2
6	Nasopharynx	1163	5.7	13.4	10.5
7	Skin (incl. Melanoma)	908	4.4	10.4	10.0
8	Lymphoma	859	4.2	9.9	9.4
9	Bladder	647	3.2	7.4	7.2
10	Leukemia	603	2.9	6.9	7.2

* CR Crude rate per 100,000 per year

** ASR Age standardised rate per 100,000 per yea. ASR Derived by the direct method using the‘World Population’.

**Table 4:** Ten most frequent cancers in females, 2001-2005

**Rank**	**Site**	**No.**	**%**	CR*	ASR**
1	Breast	6405	29.4	73.0	57.4
2	Colo-rectum	3142	14.4	35.8	29.2
3	Lung	1761	8.1	20.1	16.2
4	Ovary	1251	5.7	14.2	11.8
5	Corpus Uteri	1176	5.4	13.4	10.9
6	Cervix Uteri	1001	4.6	11.4	9.1
7	Stomach	877	4.0	10.0	7.9
8	Skin (incl. Melanoma)	798	3.7	9.1	7.2
9	Thyroid	647	3.0	7.4	6.1
10	Lymphoma	619	2.8	7.0	6.2

* CR Crude rate per 100,000 per year

** ASR Age standardised rate per 100,000 per yea. ASR Derived by the direct method using the‘World Population’.

In comparison with Western countries, Singapore has higher incidence rates for cancers of the nose, stomach and liver, and lower rates for cancers of pancreas, skin and prostate. The high incidence of colorectal cancers, which is approaching that of the West for both males and females, is due to an increasingly affluent society, with lifestyle habits such as smoking and diets rich in meat and fat being major contributing factors.

Prostate cancer is the 3^rd^ most common cancer in Singaporean males from 2001-2005, and is the number six cause of mortality amongst Singaporean males. The incidence of prostate cancer in Singaporean males is increasing, in part due to the widespread use of PSA (prostate specific antigen) as a screening test. PSA screening is commonly included in many health screening programmes.

Breast cancer is the leading cause of death in Singaporean women. Singapore has one of the highest age-adjusted breast cancer incidences in Asia with increasing incidence in women in their 50’s. Mammographic screening in Singaporean women increases the rate of detection of early breast cancers, with acceptable recall, needle biopsy rates.

Among the ethnic groups, colorectal, lung and prostate cancers were the three most common cancers among the three ethnic groups. Age standardised rates were highest among Chinese males, followed by Malay and Indian males.

For females, breast and colorectal cancers were the most common and second most common cancers respectively. The age standardised rates were highest among Chinese females, followed by Malay and Indian females. Lung cancer was the third most common among Chinese females, fourth among Malay females and eighth among Indian females.

## CURRENT MANAGEMENT OF THE TOP 3 CANCERS IN MALES AND FEMALES

The 3 top cancers among males and females are lung, colorectal and breast cancer (in females). Management entails surgery, radiotherapy or chemotherapy, either alone or in combination.

### Lung cancer

Upon suspicion of lung cancer, definitive biopsy is obtained by the diagnostic radiologist or respiratory physician via

1. Fine needle aspiration and cytology
2. Brochoscopy and biopsy

The patient is staged with a CT scan of the brain/thorax/abdomen and bone scan while a PET CT can be performed as an additional staging procedure, especially prior to surgery, to aid treatment decisions for selected patients. Tumour stage is ascribed following the 2002 AJCC staging guidelines [2]. Management is different for non-small cell lung cancer (NSCLC) and small cell lung cancer (SCLC).

For patients with **NSCLC**, surgery offers the patient with resectable disease the best chance of a cure. Surgical approaches include wedge resection, lobectomy or pneumonectomy. The type and extent of surgery performed depends on the patient’s functional status and extent of disease.

**Chemotherapy **is given concurrently with radiotherapy for patients with locally advanced disease. The agents used include cisplatin and vinblastine.

In the adjuvant setting, chemotherapy is given for patients with Stage Ib-III disease post-resection. The agents used include cisplatin, vinorelbine, carboplatin and paclitaxel.

**Radiotherapy** is an alternative for patients who are not suitable for surgery or for patients who decline surgery. Definitive radiotherapy is given for patients suitable for curative treatment, but decline surgery or chemotherapy. The dose and fractionation regimen depends on patient’s performance status / choice and treatment volume. Options include 60-70 Gy in 30-35 fractions given over 6 to 7 weeks, or 50 Gy in 20 fractions given over 4 weeks. Radiotherapy is delivered via 3-Dimensional Conformal radiotherapy (3DCRT).

Patients with stage IIIA disease are treated with Surgery and/or chemoradiotherapy depending on performance status and patients with stage IIIB disease are treated with chemoradiotherapy.

For patients who are not suitable for curative treatment or for patients with metastatic disease, palliative radiotherapy/chemotherapy or best supportive care are acceptable options. The aim is palliation of symptoms and/or prolongation of life.

**Radiotherapy techniques** such as Intensity Modulated Radiotherapy (IMRT) are available in Singapore and can be used in selected cases of lung cancer unsuitable for surgery. Local control is important in NSCLC since at least 30-40% of patients die from local or locoregional progression of disease. For curative treatment, doses in the range of 60-70 Gy are required. IMRT allows for the improvement in therapeutic ratio. A recent review from the Memorial Sloan Kettering Cancer Centre showed that IMRT resulted in promising outcomes for inoperable patients with NSCLC [3].

Currently IMRT is not the standard of care for lung cancer in Singapore, until technology for respiratory gating is available and optimised for clinical application. A carefully implemented respiratory gated IMRT programme is essential for the accurate and safe delivery of higher than conventional radiotherapy doses.

Patients with limited stage **SCLC** are managed with a combination of chemotherapy and radiotherapy.

Chemotherapy agents used include cisplatin and etoposide. Radiotherapy is given concurrently with chemotherapy, most often commencing with the 1^st^ or 2^nd^ cycle of chemotherapy. The dose of radiation used is in the order of 50-54Gy, given daily over a period of 5-6 weeks. Radiotherapy is delivered via 3DCRT. Prophylactic cranial irradiation is recommended for suitable patients with complete and good partial response after chemotherapy.

Patients with extensive stage SCLC are managed with palliative chemotherapy.

### Colorectal cancer

The diagnosis of colorectal cancer is done by colonoscopy and biopsy. Patients are staged with a CT scan of the thorax/abdomen/pelvis. A CT scan of the brain and bone scan is done if brain and bone metastasis is suspected.

The definitive treatment for colorectal cancer is surgery. While surgery alone is curative for early stage colorectal cancer, adjuvant chemotherapy and/or radiotherapy is employed in patients with locally advanced disease to reduce the risk of local recurrence and improve survival.

Surgical approaches for colon cancer includes

1. Polypectomy and local excisions, which is performed for superficial cancers or polyps.
2. Colectomy (hemicolectomy/segmental resection), which involves removing part of the colon as well as regional lymph nodes.
3. Laparoscopy-assisted colectomy, which involves removal of part of the colon as well as regional lymph nodes.

Surgical approaches for rectal cancer includes

1. Polypectomy and local excisions, which is performed for superficial cancers or polyps
2. Low anterior resection, where the tumour is removed with the regional lymph nodes with preservation of anal sphincter function
3. Abdominal perineal resection, whereby the surgeon makes 2 incisions, one in the abdomen, another in the perineum. The tumour/regional lymph nodes are removed together with the anus and sphincter muscle. Because the anus is removed, a permanent colostomy is required.
4. Pelvic exenteration is uncommonly performed for locally extensive rectal cancers. A colostomy is required and a urostomy (opening on the abdominal wall for urine to flow into a bag) is required if the bladder is also removed.

Adjuvant chemotherapy is given for patients with stage III colon cancer and also for patients with stage II colon cancer with high risk features (T4 disease, perforation of tumour, poorly differentiated tumour, positive margin). Agents used include 5 Flurouracil/leucovorin with platinum based compounds such as oxaliplatin. Other targeted agents such as cetuximab are increasingly being employed.

For colorectal cancer, adjuvant chemoradiotherapy is given for patients with stage T3-4 or node positive rectal cancer. The chemotherapy regimen used is 5 Flurouracil/leucovorin for 5 cycles with radiotherapy given concurrently with the 3^rd^ cycle of chemotherapy.

The dose of adjuvant radiotherapy prescribed is 45 Gy in 25 fractions over 5 weeks duration to the whole pelvis, followed by a 5.4 Gy in 3 fractions to boost the tumour bed. This dose is increased to 9 Gy in 5 fractions if there is a positive margin.

Preoperative chemoradiation can also be given for locally advanced rectal tumours, to attempt to down-stage the tumour. The neoadjuvant approach has been shown to lead to an increase in local control, decrease acute toxicity and increase sphincter sparing rates.

Adjuvant radiotherapy is not routinely given for colonic tumours or descending colon/sigmoid colon tumours above the peritoneal reflection because of inability to deliver radiotherapy accurately and safely to an organ that can move freely. Radiotherapy is delivered via 3DCRT.

### Breast cancer

Breast cancer is detected using mammography, or by the patient either as a palpable lump, or changes in the breast (including nipple discharge, overlying skin changes). Mammographic screening enables early detection and intervention of breast cancers, resulting in a reduction in breast cancer mortality. Breast cancer is most commonly diagnosed by a fine needle aspiration and cytology. Other approaches include trucut biopsy or excision biopsy.

**Early breast cancer** can be cured with mastectomy alone. With the advent of sentinel lymph node biopsy, a full axillary dissection can be avoided, hence avoiding the attendant complications of infection, seroma, bleeding and lymphedema.

An alternative to mastectomy is breast conservation. Breast conservation therapy entails wide local excision with axillary surgery followed by adjuvant radiotherapy to the affected breast.

The dose of adjuvant radiotherapy is 50Gy in 25 fractions to the whole breast followed by a 10Gy in 5 fraction boost to the tumour bed. A large number of randomised trials have demonstrated equivalence in long term survival compared with mastectomy with this approach.

The use of adjuvant radiotherapy following breast surgery has been shown to improve the survival of patients in clinical studies and meta-analysis.

Adjuvant radiotherapy is given using 3DCRT technique. For patients with large pendulous breast, IMRT can be used to reduce hot spots and to ensure a homogenous dose distribution, hence reducing acute and late side effects.

Adjuvant hormonal therapy is prescribed if the patient has hormone receptive breast cancer. In premenopausal patients, tamoxifen (selective estrogen receptor antagonist) is used while in postmenopausal patients, tamoxifen or an aromatase inhibitor (including Anastrozole, Letrozole, Exemestane) can be used as an alternative. The aromatase inhibitors have been shown to have less side effects such as hot flushes, endometrial cancer, vaginal bleeding, cardiovascular accidents, and thromboembolic events compared to tamoxifen. However, they are associated with an increase in arthralgia and fractures, and hence should not be used for patients with osteoporosis.

Other options of hormonal treatments include ovarian ablation with surgery or radiotherapy and ovarian function suppression with luteinising hormone-releasing hormone agonists such as goserelin.

Adjuvant chemotherapy is recommended for patients with a combination of adverse risk factors including younger patients, lymph node positivity, grade of tumour, lymphovascular invasion, Her2neu receptor positivity and tumour size.

Commonly used chemotherapy agents include anthracyclines such as doxorubicin in combination with cyclophosphamide and 5 Fluorouracil. Taxanes such as paclitaxel and docetaxel are used in patients with node positive disease, or in patients with high risk node negative disease.

**Locally advanced breast cancer **is treated with a combined modality approach. Mastectomy with an axillary dissection is performed if the tumour is surgically resectable. This is followed by adjuvant chemotherapy and adjuvant radiotherapy respectively. Adjuvant hormonal treatment is recommended for patients with hormone responsive disease.

Surgically unresectable tumours are treated with neoadjuvant chemotherapy followed by mastectomy. Adjuvant radiotherapy +/- hormonal therapy is given thereafter.

If the tumour is still unresectable after neoadjuvant chemotherapy, then radiotherapy is given first before surgery is attempted.

In patients who are unfit or decline chemotherapy, neoadjuvant hormonal therapy is an alternative to neoadjuvant chemotherapy in receptor-responsive disease.

The dose of radiotherapy given in the adjuvant setting post mastectomy is 50Gy in 25# to the chest wall over 5 weeks duration. The supraclavicular fossa and/or axilla are included in the treatment fields when indicated.

## PRESENT AND FUTURE

Since its independence in 1965, Singapore has quickly grown from being a developing country to a developed country with a population in excess of 4 million. Cancer incidence has increased and so has demands for comprehensive cancer care.

Singapore has a well-established radiotherapy service catering to the local as well as regional cancer patients. There are currently five radiotherapy centres in Singapore, two private centres and three centres based in government hospitals (which provide for both government subsidised and private cancer patients). For the public centers, the National Cancer Centre based at the Singapore General Hospital currently has 9 linear accelerators, one with Novarlis stereotactic radiosurgery/radiotherapy capabilities and one brachytherapy suite. The Cancer institute at National University Hospital and at Tan Tock Seng is equipped with 4 linear accelerators in total and one brachytherapy suite. In the private sector, the Gleneagles radiation oncology unit is equipped with two linear accelerators, and Mt Elizabeth Hospital with one linear accelerator and one state-of-the-art tomotherapy machine. Both private and public centres have established surgical and medical oncology centers to provide comprehensive cancer services.

The majority of local patients are treated at public centres. The waiting time for radiotherapy varies from a few days to as long as six weeks depending on center and urgency of treatment. In view of the aging population and the rising expectations of increasingly affluent cancer patients seeking advanced care, there are plans for growth of the cancer services in both public and private sectors. For example, a second national cancer centre with enhanced radiotherapy facilities is being built at the Kent Ridge National University Hospital campus in addition to the development of at least 2-3 new private radiotherapy centres. All of the proposed expansion plans are timed to occur in the next 4-5 years. However the expansion will also require a significant increase in staff, which will not be able to be completely covered locally. Thus recruitment for trained, experienced staff will also have to be conducted regionally. In conjunction with the increased services there will also be more emphasis placed on academic development and research particularly in the newly planned National University Cancer Institute Singapore (NCIS), as mentioned above.
